# Association between bowel movement disorders and depressive symptoms: a cross-sectional study

**DOI:** 10.3389/fpsyt.2024.1449948

**Published:** 2024-09-17

**Authors:** Linyue Wang, Maosheng Tian, Hongyuan Sun, Jihua Gao, Wenyue Qi, Jiancheng Xu, Yongkang An, Wencong Xu

**Affiliations:** ^1^ Graduate School, Hebei University of Chinese Medicine, Shijiazhuang, China; ^2^ Anorectal Surgery Department, The First Affiliated Hospital of Hebei University of Chinese Medicine, Shijiazhuang, China

**Keywords:** NHANES, depression, constipation, diarrhea, fecal incontinence

## Abstract

**Objectives:**

This study aimed to explore the association between bowel movement disorders and depression in adults.

**Method:**

A cross-sectional study was conducted using data from the National Health and Nutritional Examination Survey (NHANES), 2005–2010. Depression, constipation, diarrhea, and fecal incontinence were self-reported via questionnaires. Weighted logistic regression and subgroup analyses were performed to explore the association between bowel movement disorders and the risk of depression. Restricted cubic spline (RCS) was also conducted to investigate the association between bowel movements disorder and depression.

**Results:**

A total of 13,820 participants were collected. Compared to the participants with normal bowel movements, the full-adjusted depression model ORs for constipation and diarrhea were 2.28 (95%CI,1.78-2.92), 1.75 (95%CI,1.31-2.31), respectively. Any kind of bowel leakage were associated with depression. The RCS showed the possible nonlinear association between bowel movement frequency/stool shape and depression.

**Conclusions:**

Constipation, diarrhea, and bowel leakage are associated with an increased risk of depression.

## 1 Introduction

Depression is one of the most hazardous mental disorders, rank among the most substantial causes of death worldwide (1). In 2020, the prevalence of depression was 9.2% (2), and the incidence was increasing (3). It can increase suicide ideation, attempts, and death (4). thus, depression became the leading cause of disability around the world (5).

Defecation disorders are common clinical problems that includes constipation, diarrhea and fecal incontinence. Constipation and diarrhea are changes in bowel movement frequency and stool form, and fecal incontinence refers to the involuntary leakage of gas, mucus, liquid, or solid stool. These bowel movement disorders profoundly impair the quality of life (6–9). Evidence showed that the risk of constipation in depressed people was 1.69 to 3.76 times that of non-depressed people (10, 11). Diarrhea, the eighth leading cause of death among all ages (12), was found to be commonly comorbid with depressive symptoms (13). As for fecal incontinence, which has a worldwide prevalence of 7% and 40 newonset per 1000 person a year, can markedly impair quality of life (14, 15). Moreover, the severe symptoms of depression were thought to be an identified risk factor of fecal incontinence (15). However, insufficient large population-based studies adjusting for related variables are available to assess the association between common gastrointestinal problems and depression. In this study, we aimed to determine the relation between depression and bowel movement disorders (constipation, diarrhea, and fecal incontinence) in a nationally representative sample of US adults.

## 2 Methods

### 2.1 Study design and recruitment

Data from the National Health and Nutrition Examination Survey (NHANES) was used to conduct this study. The NHANES is a continuous cross-sectional survey of noninstitutionalized US civilians conducted by the US National Center for Health Statistics (NCHS) of the Centers for Disease Control and Prevention. Data for the NHANES was collected via household interviews, physical examinations, and laboratory data (16). For this study, bowel health data from 31,034 participants in the NHANES (2005–2010) were evaluated. The NCHS Research Ethics Review Board reviewed and approved this study. Informed consent was obtained from all subjects involved in the study.

**Figure 1:**
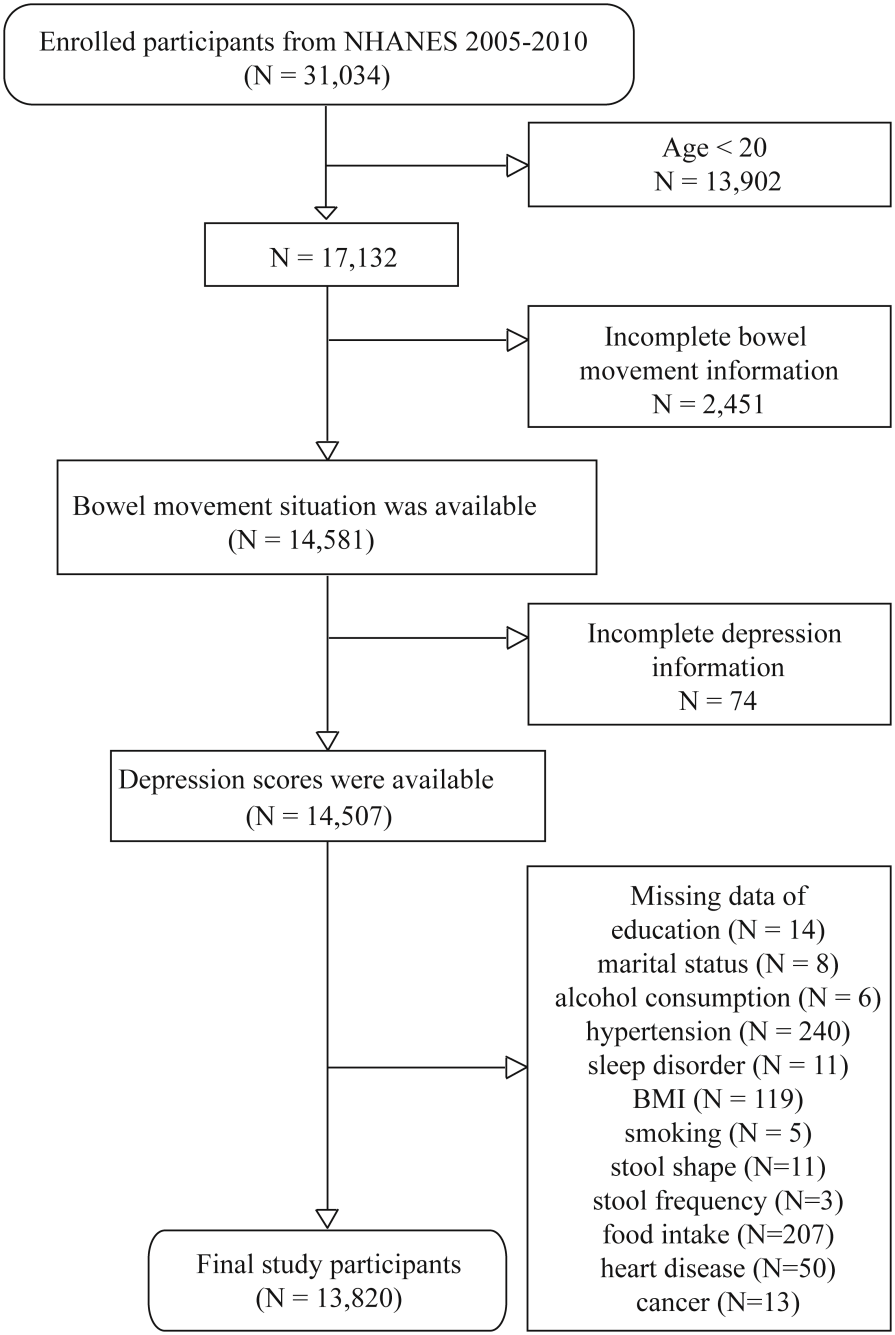
Flow chart of participants from NHANES included in the study.

Participants under 20 years old were excluded (N = 13,902) because they were not involved in the “bowel health” questionnaire. Participants with missing bowel health (N = 2,451), depression (N = 74) information and other covariate information (N = 687) were excluded. The specific screening process was shown in **Figure 1**. After the exclusion of participants, the data from 13,820 individuals were eligible for further analysis.

### 2.2 Dependent variable: depression

Depression symptoms were measured with a nine-item Patient Health Questionnaire (PHQ-9). The internal and test-retest reliabilities of the PHQ-9 were both excellent, with a Cronbach’s α of 0.89 (17). The scores of the nine items were added to calculate the depressive scores. Depression was defined as PHQ-9 scores of ≥10 (17).

### 2.3 Independent variables

Bowel movement status was evaluated using a questionnaire about weekly bowel movement frequency and the Bristol Stool Form Scale. The Bristol Stool Form Scale uses 7 descriptions of stool shape from separate hard lumps to watery stool. Constipation was defined as less than 3 bowel movements per week or 1 and 2 types of stool from the Bristol Stool Form Scale (18). Diarrhea was defined as 21 defecations per week without 1 or 2 Bristol Stool Form Scale types or Bristol Stool Form Scale type 7 stool defecation more than 2 times per week (19).

Fecal incontinence was defined as the unintentional loss of solid or liquid stool. The Fecal Incontinence Severity Index (FISI) consists of 4 questions about the frequency of different types of bowel leakage, including gas, mucus, liquid, and solid stool. The answer choices represented the frequency of leakage and included “1” for “2 or more times a day,” “2” for “once a day,” “3” means “2 or more times a week,” “4” for “once a week,” “5” for “1 to 3 times a month,” and “6” for “never.” It assigns a cumulative subjective weighted score from 0 to 61 to each patient, where a value of ‘0’ indicates no incontinence and ‘61’ indicates incontinence to gas, liquid, mucus and solid stool at least twice daily (20). Fecal incontinence was defined as any type of leakage on the FISI (21).

### 2.4 Covariates

Sociodemographic information (age, gender, marital status, ethnicities, and education), lifestyles (smoking, alcohol drinking, and sleep situation), medical conditions (hypertension, heart disease, cancer), antidepressant use, food intake (energy intake, protein intake, dietary fiber, and carbohydrate consumption), and body mass index (BMI) were collected. Age was divided into four categories: 20–39, 40–59, 60–79, and 80 and above. Marital status was classified into two groups: married/live with partner and widowed/divorced/separated/never married. Educational levels were classified into two categories: high school or below and above high school. Participants were considered smokers if they chose “YES” on the questionnaire: “Have you smoked at least 100 cigarettes in your entire life?” and also chose “Every day” or “Some days” on the questionnaire: “Do you now smoke cigarettes”. Alcohol users were defined if they consumed at least 12 drinks in any one year. Sleep disorder was defined as a participant complaint to the doctor about trouble sleeping or the participant was told by a doctor that they had a sleep disorder. Hypertension was defined as a high systolic (≥130 mmHg) or diastolic (≥80 mmHg) blood pressure measured at the mobile examination center, or self-report history of hypertension, or use of antihypertension medication. Participants were considered heart disease as if they chose “YES” on the questionnaire: “Ever told had congestive heart failure?” or “Ever told you had coronary heart disease?”. Cancer participants were defined if they had ever been told having cancer or a malignancy. Antidepressants were identified by the second level codes 249 in NHANES Drug Information (RXQ_DRUG). The list of antidepressants can be found in **Supplementary Table 1**. The energy, protein, fiber, and carbohydrate intake data were obtained from total nutrient intakes data by taking the average of two-day-recall. BMI was calculated as weight in kilograms divided by height in meters squared and was categorized into four groups (22): under-weight (BMI<18.5), normal (18.5≤ BMI≤ 25) overweight (25<BMI<30), and obesity (BMI≥30).

### 2.5 Statistical analysis

All statistical analyses accounted for the complex sampling design of the NHANES database, sample weights of individuals were determined by WTDR2D/3. Categorical variables are presented as weighted percentages and analyzed with Chi-square tests. Multivariable logistic regression models were used to estimate odds ratios (ORs) and 95% confidential intervals (CIs) for associations between depression and defecation disorders. Model 1 was not adjusted for any variables; Model 2 was adjusted for age, gender, race; Model 3 was adjusted for the variables in Model 2 plus education levels, marital status, BMI, sleep status, alcohol use, smoking, hypertension, heart disease, cancer, antidepressant use, and food intake (energy intake, protein intake, dietary fiber, and carbohydrate consumption). Subgroup analyses were stratified by age, gender, BMI to assess the association of depression with defecation problems. Meanwhile, interaction analyses were conducted to assess potential interactions. Furthermore, a restricted cubic spline (RCS) curve was performed to investigate the potentially nonlinear association between depression and defecation disorders. If the RCS showed a nonlinear relation, its inflection point would be further calculated. Sensitivity assessments were performed to evaluate the robustness of the results by conducting unweighted logistic regression analysis, modifying adjusted factors, and re-run the same models with depression defined as PHQ-9 scores ≥ 8. Statistical analysis was performed using Stata software (version 16.0) and R version 4.1.1. Two-tailed tests were conducted and a *P* value less than 0.05 was considered statistically significant.

## 3 Result

### 3.1 Baseline characteristics of participants

The study included 13,820 participants representing 191,491,012 civilian adults in the USA. 3,212 participants whom we failed to interview were excluded. **Supplementary Table 2** displayed the baseline characteristics of adults with or without depression in the 2005-2010 NHANES. There were 1,182 (weighted n =14,520,742) participants had depression. Compared to non-depression participants, they were more likely to be 40 to 59 years old non-Hispanic White females, to have lower education levels, to be current smokers and drinkers, to have a higher BMI, to have higher prevalence of hypertension and heart disease, to have lower energy and protein intake, and to have higher prevalence of fecal leakage or abnormal bowel movements (*P*<0.05).

### 3.2 Association between depression and abnormal defecation

Depression positively correlated with defecation problems (**Table 1**). Compared to participants with normal bowel movement habits, participants with constipation had increased odds of significant depression (OR (95% CI) = 2.78 (2.19, 3.53)) in the unadjusted model (Model 1); the OR was 2.28 (95% CI, 1.78, 2.92) after full adjustment (Model 3). Compared to participants with normal bowel movement habits, participants with diarrhea had increased odds of depression (OR (95% CI) = 1.84 (1.37, 2.47)) in the unadjusted model and after full adjustment (OR (95% CI) = 1.74 (1.31, 2.31)).

**Table 1:** Association of depression with bowel movement problems in a US population: NHANES, 2005–2010.

Exposure	Model 1		Model 2		Model 3	
	OR (95%CI)	*P*	OR (95%CI)	*P*	OR (95%CI)	*P*
Bowel Movements						
Constipation	2.78(2.19,3.53)	<0.001	2.48(1.96,3.16)	<0.001	2.28(1.78,2.92)	<0.001
Diarrhea	1.84(1.37,2.47)	<0.001	1.93(1.44,2.60)	<0.001	1.74(1.31,2.31)	<0.001
*P* for trend	<0.001		<0.001		<0.001	
Fecal Incontinence						
Gas	1.25(1.00,1.56)	0.049	1.25(1.01,1.55)	0.042	1.27(1.01,1.59)	0.042
Mucus	4.00(2.18,7.32)	<0.001	4.17(2.28,7.63)	<0.001	3.49(1.63,7.46)	0.002
Fluid	3.51(2.67,4.62)	<0.001	3.98(2.97,5.32)	<0.001	3.22(2.38,4.35)	<0.001
Solid	2.57(1.81,3.64)	<0.001	2.93(1.98,4.34)	<0.001	2.25(1.55,3.26)	<0.001
*P* for trend	<0.001		<0.001		<0.001	

Model 1: Unadjusted.

Model 2: Adjusted for age, sex, ethnicity.

Model 3: Adjusted for age, sex, ethnicity, education, marital status, BMI, sleep status, alcohol, smoking and hypertension, heart disease, cancer, antidepressant usage, and food intake (energy, fiber, carbon, and protein).

In the fully adjusted Model 3, all categories of bowel leakage showed significant positive association with the incidence of depression (OR (95% CI): 1.27 (1.01, 1.59), OR (95% CI):3.49 (1.63, 7.46), OR (95% CI): 3.22 (2.38, 4.35), and OR (95% CI): 2.25(1.55, 3.26), respectively), as presented in **Table 2**.

**Table 2:** Stratified analysis of association between abnormal bowel movements and depression.

Subgroup	Constipation		Diarrhea		*P* for interaction
	OR (95%CI)	*P*-value	OR (95%CI)	*P*-value	
**Gender**					0.47
Male	2.40(1.31,4.39)	<0.01	1.58(1.08,2.30)	0.02	
Female	2.18(1.64,2.88)	<0.01	1.97(1.43,2.73)	<0.01	
**Age**					0.33
20~39	1.90(1.29,2.80)	<0.01	1.47(0.84,2.56)	0.17	
40~59	2.93(1.92,4.48)	<0.01	1.89(1.27,2.81)	<0.01	
60~79	1.82(1.03,3.22)	0.04	1.83(0.88,3.83)	0.11	
80 and over	0.80(0.23,2.81)	0.72	2.61(0.69,9.86)	0.15	
**BMI**					0.70
Normal	2.55(1.59,4.10)	<0.01	1.54(0.73,3.25)	0.25	
Under-weight	0.15(0.01,1.78)	0.13	4.31(0.22,83.38)	0.33	
Overweight	2.38(1.56,3.61)	<0.01	2.04(1.13,3.66)	0.02	
Obesity	2.10(1.40,3.13)	<0.01	1.65(1.13,2.42)	0.01	

Adjusted for age, sex, ethnicity, education, marital status, BMI, sleep status, alcohol, smoking and hypertension, heart disease, cancer, antidepressant usage, and food intake (energy, fiber, carbon, and protein).

### 3.3 Subgroup analysis

**Figure 2:**
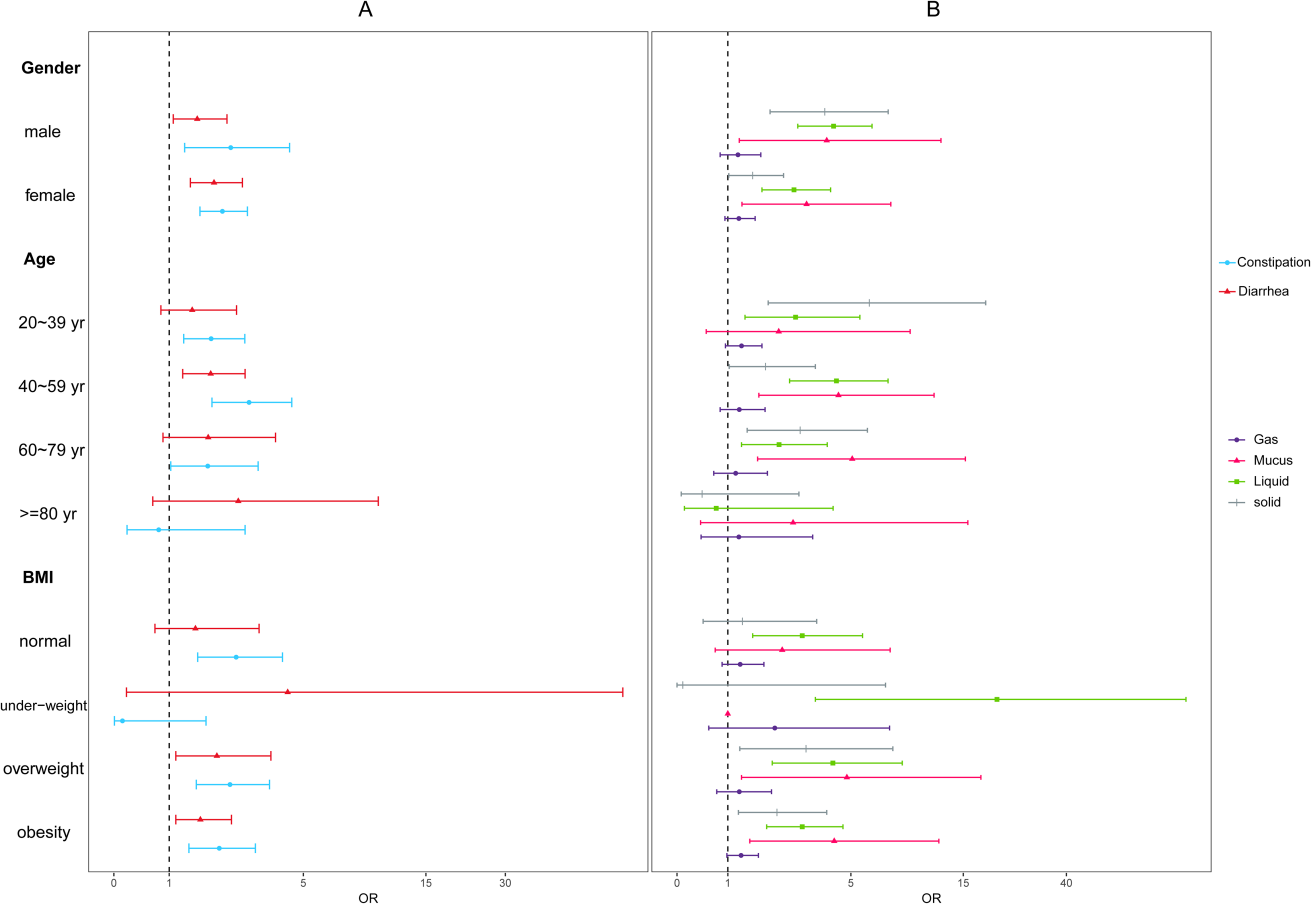
Subgroup analysis adjusted for age, sex, ethnicity, education, marital status, BMI, sleep status, alcohol, smoking and hypertension, heart disease, cancer, antidepressant usage, and food intake (energy, fiber, carbon, and protein) **(A)**. Subgroup analysis of association between bowel movement and depression; **(B)**. Subgroup analysis of association between fecal incontinence and depression.

Subgroup analysis of association between bowel movements and depression was conducted according to fully adjusted multivariate logistic regression stratified by age, gender, and BMI (**Table 2**, **Figure 2A**). According to stratified analysis results, we discovered for those who were under 80 years old, overweight or obesity, constipation and diarrhea might increase the depression risk. The interaction test did not find any statistically significant differences among all subgroups. (*P* for interaction > 0.05).

**Table 3** and **Figure 2B** showed the stratified analysis of association between fecal incontinence and depression. The result indicated that for those who were 40 to 59 year-old overweight or obesity participants, mucus, liquid and solid bowel leakage might increase the depression risk. For those with normal BMI, only liquid leakage would increase the depression risk (OR (95% CI): 2.97(1.56,5.63)). In addition, the interaction test did not find any statistically significant differences among all subgroups. (*P* for interaction > 0.05).

**Table 3:** Stratified analysis of association between fecal incontinence and depression.

Subgroup	Gas		Mucus		Liquid		Solid		*P* for interaction
	OR (95%CI)	*P*	OR (95%CI)	*P*	OR (95%CI)	*P*	OR (95%CI)	*P*	
**Gender**									0.09
Male	1.22(0.84,1.76)	0.28	3.89(1.25,12.11)	0.02	4.18(2.82,6.19)	<0.01	3.81(2.00,7.26)	<0.01	
Female	1.24(0.94,1.62)	0.12	3.12(1.31,7.45)	<0.01	2.70(1.79,4.05)	<0.01	1.56(1.02,2.38)	<0.01	
**Age**									0.05
20~39	1.30(0.95,1.79)	0.10	2.24(0.56,9.00)	0.25	2.75(1.38,5.48)	0.01	6.02(1.95,18.57)	<0.01	
40~59	1.25(0.84,1.87)	0.27	4.40(1.71,11.34)	<0.01	4.31(2.56,7.25)	<0.01	1.88(1.03,3.44)	<0.01	
60~79	1.17(0.71,1.93)	0.52	5.07(1.68,15.30)	0.01	2.25(1.30,3.91)	0.01	2.90(1.43,5.91)	<0.01	
80 and over	1.24(0.46,3.34)	0.66	2.67(0.45,15.67)	0.27	0.76(0.14,4.16)	0.75	0.48(0.08,2.86)	0.41	
**BMI**									0.33
Normal	1.27(0.88,1.84)	0.20	2.34(0.74,7.40)	0.15	2.97(1.56,5.63)	<0.01	1.32(0.50,3.49)	0.56	
Under-weight	2.13(0.61,7.36)	0.23	NA	NA	20.68(3.44,124.29)	<0.01	0.11(0.00,7.07)	0.29	
Overweight	1.25(0.77,2.04)	0.36	4.80(1.30,17.74)	0.02	4.15(2.06,8.33)	<0.01	3.10(1.26,7.60)	0.02	
Obesity	1.29(0.98,1.70)	0.06	4.21(1.49,11.87)	0.01	2.97(1.91,4.61)	<0.01	2.19(1.23,3.89)	0.01	

Adjusted for age, sex, ethnicity, education, marital status, BMI, sleep status, alcohol, smoking and hypertension, heart disease, cancer, antidepressant usage, and food intake (energy, fiber, carbon, and protein).

The NA means “Not Available”.

### 3.4 Non-linearity analysis between abnormal defecation and depression

**Figure 3:**
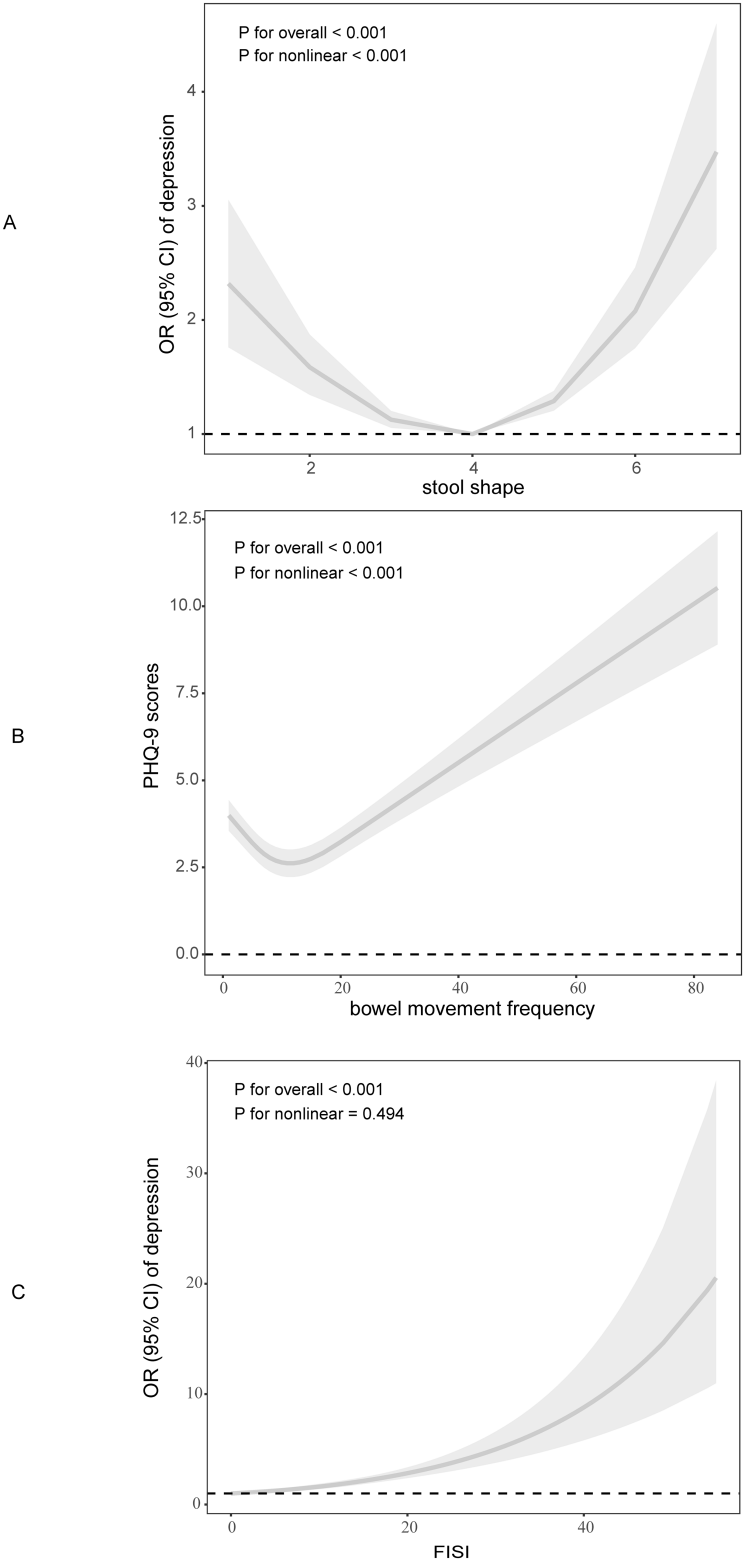
Dose-response connection of defecation disorders with depression based on full adjusted model. **(A)**. Stool shape and depression; **(B)**. Bowel movements frequency and PHQ-9 scores; **(C)**. FISI and depression.

We used restricted cubic spline (RCS) plots to explore the non-linear association of defecation disorders and depression (**Figure 3**). We built three RCS plots according to Model 3 by gender stratification. **Figure 3A** showed a ‘U-shape’ curve between the stool shape and depression (*P* for non-linear <0.05). Only when the stool shape represented as “like a sausage or snake, smooth and soft” (23), the odd ratio of depression was not increased. Furthermore, we found that overall inflection point was 3. Significant associations were found with the ORs (95% CI) of 0.66 (0.57, 0.77) after the inflection point and with the ORs (95% CI) of 1.23 (0.14, 1.32) before the inflection point (**Table 4**).

**Table 4:** Analysis of the threshold effect implementing the two-piecewise linear regression model.

	Stool shape and depression		Stool frequency and PHQ-9 scores		FISI and depression	
	OR (95%CI)	*P* value	β (95%CI)	*P* value	OR (95%CI)	*P* value
Inflection point (K)	3		6		10	
< K	0.66(0.57,0.77)	<0.01	-0.36(-0.43,-0.30)	<0.01	1.05(1.03 ,1.06)	<0.01
> K	1.23(1.14,1.32)	<0.01	0.04(0.02,0.05)	<0.01	1.07(1.05,1.08)	<0.01
*P* for likelihood ratio	<0.01		<0.01		0.20	

We further analyzed non-linear association between the bowel movement frequency and depression PHQ-9 scores (**Figure 3B**). The result indicated that there was a ‘J-shape’ curve between them (*P* for non-linear <0.05). The inflection point was 6 (**Table 4**). For those who have less than 6 bowel movements per week, the frequency of bowel movements was negatively associated with PHQ-9 scores, and positively associated with more than six bowel movements per week.

In **Figure 3C**, we illustrated the linear association between FISI scores and depression (*P* for non-linear > 0.05). High FISI scores are accompanied by a higher risk of depression.

### 3.5 Sensitivity assessments

Using unweighted logistic analysis indicated the similar results with previous analysis. Constipation, diarrhea, and fecal incontinence were all associated with depression (details in **Supplementary Table 3**). Then, we modified the adjusted factors to evaluate the robustness of the results. After adjusted for age, sex, ethnicity, education, marital status, BMI, sleep status, alcohol, smoking, antidepressant usage, and food intake in Model 3, the association between depression and bowel movement disorders remained (details in **Supplementary Table 4**). Furthermore, the **Supplementary Table 5** showed the sensitivity analysis by re-defined depression as PHQ-9 score ≥8. The bowel movements were still related with depression. All the sensitivity analysis results represented that the association between bowel movement disorders and depression was stable.

## 4 Discussion

This study investigated the association between defecation disorders and the risk of depression in adults based on data from 2005 to 2010 NHANES. The results showed that constipation, diarrhea, and fecal incontinence had positive relations with the risk of depression after adjusting for potential factors. Of all the types of fecal incontinence, that of mucus is most likely to be combined with the presence of depression(OR (95%CI): 3.49 (1.63, 7.46)). The interaction test did not find any statistically significant differences among gender, age and BMI subgroups. Moreover, there was a significant “U-shaped” dose–response relation between the stool shape and the risk of depression. When the stool shape representing “sausage-like” with cracks on its surface or smooth and soft shape, the risk of depression was the lowest. A “J-shaped” dose–response relation between the stool frequency and the PHQ-9 scores was founded. Six bowel movements per week was considered to be the optimal frequency for lower PHQ-9 scores, in which case the risk of depression is lower. Our results indicated that the abnormal defecation was positively associated with the risk of depression.

Several studies have highlighted the link between constipation and depression. On one hand, a study showed that high anxiety level was the independent factors for predicting the perception of constipation (24). On the other hand, constipation might lead to a high risk of depressive symptoms. Shatri (25) found chronic functional constipation patients were commonly companied with depressive symptoms. Another study of a total of 1870 sample showed that constipation severely altered the participants’ quality of life (26). In addition, research showed the suicide mortality of acutely admitted patients was related to gastrointestinal disorders, especially constipation (27). Although there were previous studies from NHANES data illustrating that people with constipation and diarrhea were more likely to be found in depressed populations (7, 11), the data included was only one cycle of data from 2009-2010, whereas the present study included data from three cycles to make the results more convincing. Last year, Wang P et al. (28) published a study to illustrate the constipation was significantly associated with depression through NHANES 2005-2010. Of his results, the association between constipation and depression was not affected by age in both layers (<65 years old and ≥65 years old). However, our study divided age into four levels, and the results showed that the association was not significant in people aged 80 years and above. Besides, Wang P et al.’s study showed that there was no statistically significant association between constipation and depression in male, whereas our findings showed that both male and female, constipation and depression was associated. The discrepancy between the two studies may be due to different definitions of constipation and different adjustment factors. In addition, we consolidated data on fecal incontinence. Patients with fecal incontinence and concurrent constipation had poor overall quality of life (29) due to the stigma associated with fecal incontinence (30), which was consistent with our findings. In our study, diarrhea also increased the risk of depression for patients under 80 years old with BMI over 25 kg/m^2^, which was similar to Lu’s study (31).

To the best of our knowledge, this was the first study to explore the non-linear association between bowel movement and depression in a sizeable and complex cohort of American adults. Long L (32) found bowel movements frequency indicated the prognosis of hepatocellular carcinoma. Compared to those with daily bowel movements, participants with bowel movement more than once per day had a hazard ratio of 1.93. Another study illustrated the relationship between bowel frequency and cardiovascular disease and mortality of female. The result showed that compared with daily bowel movement, having bowel movements more than once daily was significantly associated with increased risk of cardiovascular disease (hazard ratio: 1.13) and total mortality (hazard ratio: 1.17) (33). Our study showed six times bowel movements per week corresponded to the lowest risk of depression, which was generally in line with the findings of the above studies. The non-linear association between stool shape and depression risk indicated type 3 of stool shape had the lowest risk of depression, in accordance with previous studies, fecal types 3, 4 and 5 were considered the most ‘normal’ stool shape (34).

The potential mechanisms behind the association between defecation problems and depression are unknown. However, the brain-gut axis may be involved in the association of diarrhea and constipation with depressive symptoms. Serotonin (5-HT) is an important modulator of microbiota-gut-brain axis. Zhou et al. found significant differences in gut microbiota between depressed patients and healthy controls. Transplanting fecal microbiota from healthy participants into depressed mice alleviated depressive symptoms by increasing 5-HT levels in the brain and colon of the mice (35). 5-HT interacts with receptors on the intestinal nervous system to modulate gut motility and to induce further signaling on emotion-regulating brain networks (36). Defects in the neural production of 5-HT might result in both mood disorders and intestinal dysfunction (37). Evidence showed that modulating the gut microbiome can alleviate depressive symptoms by regulating 5-HT metabolism (38). The correlations of behaviors and common metabolites showed that choline, betaine, and glycine metabolites were significantly associated with constipation and depression (39). In addition, patients with diarrhea-predominant irritable bowel syndrome were found have similar alterations in fecal microbiota with depression (40). Altogether, these studies suggest that the cooccurrence of depression and bowel disorders disturbs the gut microbiome. The peripheral signals from the microbiota may modulate the central processes causing depressive symptoms. Recent studies showed that probiotics could relieve the bowel movements (9, 41) and improve depressive symptoms (42–45), which is consistent with the point above. As for individuals with fecal incontinence, Cichowski et al. (46) found that women with fecal incontinence experienced more dyspareunia and fear than women without fecal incontinence. The unconscious bowel leakage might produce a feeling of being isolated, resulting in psychological and depressive symptoms (47, 48).

Several shortcomings of this study should be considered. First, the effects of medications were not considered in this study. Some antidepressants can lead to constipation (49) or diarrhea (50). Secondly, constipation and diarrhea are two general symptoms. Although the criteria for constipation and diarrhea were credible, the subtypes (for example, slow transit constipation or obstructive constipation) were not considered. Thirdly, bias was inevitable in cross-sectional studies, a large cohort study for further accurate evidence is still needed.

In summary, this study suggests that constipation, diarrhea, and fecal incontinence predict a higher risk of depression among adults. Further studies with bowel movement management and a prospective study design are warranted to confirm our observations.

## Data Availability

The original contributions presented in the study are included in the article/**Supplementary Material**, further inquiries can be directed to the corresponding author.
