# Supplementary material for: Association between bowel movement disorders and depressive symptoms: a cross-sectional study
Source: Front Psychiatry. 2024 Sep 17;15:1449948. doi: 10.3389/fpsyt.2024.1449948 (PMC11442234; doi:10.3389/fpsyt.2024.1449948)
Supplement: Supplementary file 1 [file Table1.docx]

Supplementary Material

# Supplementary Table 1

Table S1 List of antidepressants and their classifications

| **The second level code** | **The third level code/Drug classification** | **Medication name** |
| --- | --- | --- |
| 249 - ANTIDEPRESSANTS | 250 - Monoamine oxidase inhibitors | SELEGILINE |
|  | 306 - Phenylpiperazines | TRAZODONE  NEFAZODONE |
|  | 308 - Serotonin and norepinephrine reupdate inhibitors | VENLAFAXINE  DULOXETINE  MILNACIPRAN  DESVENLAFAXINE |
|  | 208 - Selective serotonin reuptake inhibitors | FLUOXETINE  SERTRALINE  PAROXETINE  FLUVOXAMINE  CITALOPRAM  ESCITALOPRAM |
|  | 209 - Tricyclic antidepressants | SEROTONIN  NORTRIPTYLINE  DESIPRAMINE  AMITRIPTYLINE  DOXEPIN  IMIPRAMINE  PROTRIPTYLINE |
|  | 307 - Tetracyclic antidepressants | NA |
|  | 76 - Miscellaneous antidepressants | BUPROPION |

# Supplementary Table 2

Table S2 Group differences between depression and non-depression groups.

| **Variables** | **Non-Depression** | **Depression** | ***P-*value** |
| --- | --- | --- | --- |
| **N(weighted)** | 12890(167,795,863) | 1214(12,782,943) |  |
| **Gender (%)** |  |  | <0.001 |
| Male | 49.66(48.95,50.38) | 35.99(33.07,39.01) |  |
| Female | 50.34(49.62,51.05) | 64.01(60.99,66.93) |  |
| **Age (%)** |  |  | <0.001 |
| 20~39 | 37.09(35.48,38.72) | 35.31(32.16,38.59) |  |
| 40~59 | 38.99(37.79,40.20) | 48.43(45.08,51.80) |  |
| 60~79 | 20.22(18.81,21.71) | 14.56(12.74,16.60) |  |
| 80 and over | 3.71(3.23,4.25) | 1.70(1.15,2.51) |  |
| **Ethnicity (%)** |  |  | <0.001 |
| Mexican American | 8.05(6.43,10.04) | 8.99(6.31,12.65) |  |
| Other Hispanic | 4.19(3.13,5.58) | 6.58(4.39,9.76) |  |
| Non-Hispanic White | 71.89(68.09,75.39) | 64.01(57.61,69.95) |  |
| Non-Hispanic Black | 10.54(8.81,12.56) | 15.95(12.92,19.53) |  |
| Others | 5.33(4.50,6.30) | 4.47(3.07,6.47) |  |
| **Education (%)** |  |  | <0.001 |
| High school or lower | 41.18(38.82,43.58) | 58.29(53.54,62.89) |  |
| More than high school | 58.82(56.42,61.18) | 41.71(37.11,46.46) |  |
| **Marital status (%)** |  |  | <0.001 |
| Married, living with partner | 66.53(64.77,68.25) | 51.33(47.74,54.92) |  |
| Widowed, divorced, separated or never married | 33.47(31.75,35.23) | 48.67(45.08,52.26) |  |
| **alcohol (%)** |  |  | 0.033 |
| No | 23.66(21.83,25.59) | 26.99(24.29,29.86) |  |
| Yes | 76.34(74.41,78.17) | 73.01(70.14,75.71) |  |
| **Fecal incontinence (%)** | |  | <0.001 |
| No | 53.33(51.62,55.03) | 41.25(37.34,45.27) |  |
| Yes | 46.67(44.97,48.38) | 58.75(54.73,62.66) |  |
| gas leakage | 39.32(37.71,40.96) | 38.49(34.69,42.43) |  |
| mucus leakage | 1.28(1.04,1.58) | 3.72(2.35,5.83) |  |
| fluid leakage | 4.48(4.01,4.99) | 12.88(10.81,15.29) |  |
| solid leakage | 1.59(1.35,1.87) | 3.66(2.72,4.9) |  |
| **Bowel movement (%)** | |  | <0.001 |
| Normal | 85.22(84.39,86.01) | 71.71(68.61,74.63) |  |
| Constipation | 8.84(8.19,9.53) | 18.98(15.99,22.38) |  |
| Diarrhea | 5.95(5.47,6.46) | 9.30(7.62,11.32) |  |
| **Hypertension (%)** |  |  | <0.001 |
| No | 50.79(49.21,52.37) | 42.01(38.73,45.36) |  |
| Yes | 49.21(47.63,50.79) | 57.99(54.64,61.27) |  |
| **Sleep disorder (%)** |  |  | <0.001 |
| No | 76.29(75.24,77.31) | 39.35(35.71,43.1) |  |
| Yes | 23.71(22.69,24.76) | 60.65(56.9,64.29) |  |
| **Smoke (%)** |  |  | <0.001 |
| No | 79.15(77.88,80.37) | 57.21(53,61.31) |  |
| Yes | 20.85(19.63,22.12) | 42.79(38.69,47) |  |
| **BMI (%)** |  |  | <0.001 |
| Normal | 30.00(28.54,31.5) | 25.46(21.98,29.28) |  |
| Under-weight | 1.61(1.34,1.95) | 1.41(0.61,3.23) |  |
| Overweight | 33.98(32.83,35.16) | 28.50(25.69,31.5) |  |
| Obesity | 34.41(32.87,35.97) | 44.63(40.37,48.96) |  |
| **Energy (%)** |  |  | <0.001 |
| Quartile 1 | 21.01(19.89,22.18) | 29.83(25.74,34.28) |  |
| Quartile 2 | 25.07(24.05,26.13) | 25.57(21.39,28.06) |  |
| Quartile 3 | 26.16(24.99,27.37) | 23.56(20.90,26.45) |  |
| Quartile 4 | 27.75(26.50,29.04) | 22.03(19.59,24.69) |  |
| **Protein (%)** |  |  | <0.001 |
| Quartile 1 | 20.93(19.82,22.10) | 34.83(30.02,39.98) |  |
| Quartile 2 | 24.61(23.65,25.60) | 23.64(20.39,27.22) |  |
| Quartile 3 | 25.88(24.52,27.28) | 20.82(18.30,23.59) |  |
| Quartile 4 | 28.58(27.30,29.89) | 20.71(17.51,24.31) |  |
| **Carbon (%)** |  |  | 0.033 |
| Quartile 1 | 22.91(21.81,24.05) | 26.30(22.18,30.89) |  |
| Quartile 2 | 25.63(24.62,26.66) | 28.33(24.72,32.24) |  |
| Quartile 3 | 25.61(24.77,26.46) | 23.29(19.67,27.34) |  |
| Quartile 4 | 25.86(24.51,27.25) | 22.08(19.31,25.13) |  |
| **Fiber (%)** |  |  | <0.001 |
| Quartile 1 | 22.32(20.78,23.93) | 36.42(32.10,40.98) |  |
| Quartile 2 | 24.20(23.32,25.11) | 27.10(23.83,30.65) |  |
| Quartile 3 | 26.29(25.25,27.37) | 20.43(17.20,24.10) |  |
| Quartile 4 | 27.19(25.50,28.94) | 16.04(13.42,19.07) |  |
| **Heart disease (%)** |  |  | <0.001 |
| No | 95.69(95.14,96.18) | 92.62(90.61,94.23) |  |
| Yes | 4.31(3.82,4.86) | 7.38(5.77,9.39) |  |
| **Cancer (%)** |  |  | 0.057 |
| No | 90.98(90.11,91.77) | 87.88(84.98,90.28) |  |
| Yes | 9.03(8.23,9.89) | 12.12(99.72,15.02) |  |
| **Antidepressant (%)** |  |  | <0.001 |
| No | 90.12(89.23,90.94) | 69.32(65.06,73.28) |  |
| Yes | 9.88(9.06,10.77) | 30.68(26.72,34.94) |  |

% for categorical variables: the p-value was calculated by a weighted chi-square test. BMI, body mass index.

# Supplementary Table 3

Table S3 Sensitivity analysis: Unweighted logistic regression analysis on the association between depression and bowel movement disorder

|  | **Model 1** | | **Model 2** | | **Model 3** | |
| --- | --- | --- | --- | --- | --- | --- |
| Exposure | OR (95%CI) | *P* | OR (95%CI) | *P* | OR (95%CI) | *P* |
| **Bowel Movements** |  |  |  |  |  |  |
| Constipation | 2.26(1.93,2.66) | <0.001 | 2.00(1.70,2.35) | <0.001 | 1.85(1.55,2.22) | <0.001 |
| Diarrhea | 2.01(1.65,2.44) | <0.001 | 2.08(1.71,2.53) | <0.001 | 1.86(1.51,2.30) | <0.001 |
| *P* for trend | <0.001 |  | <0.001 |  | <0.001 |  |
| **Fecal Incontinence** |  |  |  |  |  |  |
| Gas | 1.37(1.19,1.56) | <0.001 | 1.35(1.18,1.55) | <0.001 | 1.37(1.19,1.58) | <0.001 |
| Mucus | 3.50(2.50,4.91) | <0.001 | 3.57(2.54,5.03) | <0.001 | 3.28(2.27,4.74) | <0.001 |
| Fluid | 3.84(3.14,4.70) | <0.001 | 4.28(3.48,5.26) | <0.001 | 3.40(2.72,4.26) | <0.001 |
| Solid | 3.77(2.81,5.06) | <0.001 | 4.33(3.20,5.86) | <0.001 | 3.30(2.36,4.60) | <0.001 |
| *P* for trend | <0.001 |  | <0.001 |  | <0.001 |  |

Model 1: Unadjusted.

Model 2: Adjusted for age, sex, ethnicity.

Model 3: Adjusted for age, sex, ethnicity, education, marital status, BMI, sleep status, alcohol, smoking , hypertension, heart disease, cancer, antidepressant usage, and food intake (energy, fiber, carbon, and protein).

# Supplementary Table 4

Table S4 Sensitivity analysis: Modified full-adjusted model

| Model 3 | Bowel Movements | | Fecal Incontinence | | | |
| --- | --- | --- | --- | --- | --- | --- |
|  | Constipation | Diarrhea | Gas | Mucus | Fluid | Solid |
| OR  (95%CI) | 2.29  (1.79,2.92) | 1.76  (1.34,2.33) | 1.28  (1.02,1.61) | 3.59  (1.69,7.64) | 3.34  (2.50,4.46) | 2.40  (1.67,3.44) |
| *P* | <0.001 | <0.001 | 0.035 | 0.001 | <0.001 | <0.001 |

Model 3: Adjusted for age, sex, ethnicity, education, marital status, BMI, sleep status, alcohol, smoking, antidepressant usage, and food intake (energy, fiber, carbon, and protein).

# Supplementary Table 5

Table S5 Sensitivity analysis: The association between depression (PHQ-9 ≥ 8) and bowel movement disorders

|  | Model 1 | | Model 2 | | Model 3 | |
| --- | --- | --- | --- | --- | --- | --- |
| Exposure | OR (95%CI) | *P* | OR (95%CI) | *P* | OR (95%CI) | *P* |
| **Bowel Movements** |  |  |  |  |  |  |
| Constipation | 2.65(2.21,3.18) | <0.001 | 2.32(1.94,2.78) | <0.001 | 2.13(1.79,2.55) | <0.001 |
| Diarrhea | 1.80(1.31,2.45) | 0.001 | 1.91(1.39,2.62) | <0.001 | 1.75(1.28,2.38) | 0.001 |
| *P* for trend | <0.001 |  | <0.001 |  | <0.001 |  |
| **Fecal Incontinence** |  |  |  |  |  |  |
| Gas | 1.31(1.11,1.55) | 0.002 | 1.31(1.11,1.54) | 0.002 | 1.34(1.13,1.59) | 0.001 |
| Mucus | 3.92(2.43,6.33) | <0.001 | 4.14(2.60,6.59) | <0.001 | 3.69(2.16,6.28) | <0.001 |
| Fluid | 3.38(2.52,4.53) | <0.001 | 3.86(2.82,5.29) | <0.001 | 3.27(2.37,4.51) | <0.001 |
| Solid | 2.55(1.76,3.70) | <0.001 | 2.93(1.95,4.39) | <0.001 | 2.37(1.62,3.47) | <0.001 |
| *P* for trend | <0.001 |  | <0.001 |  | <0.001 |  |

Model 1: Unadjusted.

Model 2: Adjusted for age, sex, ethnicity.

Model 3: Adjusted for age, sex, ethnicity, education, marital status, BMI, sleep status, alcohol, smoking, hypertension, heart disease, cancer, antidepressant usage, and food intake (energy, fiber, carbon, and protein).
